# Atlanta Medical College

**Published:** 1872-10

**Authors:** 


The Atlanta Medical College.—The exercises of this institution were opened at the time announced, by an elegant address, introductory to the course, by Dr. A. W. Griggs, Professor of the Principles and Practice of Medicine. We were pleased to learn that the number of students in attendance at the opening of the present course would double the number present at the commencement of any course since the war. We are informed by the Faculty that they confidently expect the present class to reach at least one hundred students.

